# The Participation of Senior Citizens in Policy-Making: Patterning Initiatives in Europe

**DOI:** 10.3390/ijerph18010034

**Published:** 2020-12-23

**Authors:** Roberto Falanga, Andreas Cebulla, Andrea Principi, Marco Socci

**Affiliations:** 1 Instituto de Ciências Sociais, Universidade de Lisboa, 1600-189 Lisbon, Portugal; roberto.falanga@ics.ulisboa.pt; 2 Australian Industrial Transformation Institute, Flinders University, GPO Box 2100, Adelaide, SA 5001, Australia; andreas.cebulla@flinders.edu.au; 3 Centre for Socio-Economic Research on Aging, IRCCS INRCA—National Institute of Health and Science on Aging, 60124 Ancona, Italy; m.socci@inrca.it

**Keywords:** citizen participation, policy-making, senior citizens, Europe

## Abstract

Worldwide, active aging policy calls for greater participation of senior citizens in the social, economic, and political realms. Despite emerging evidence of initiatives engaging senior citizens in social activities, little is known about the use of participatory approaches in the design and/or implementation of policies that matter to older citizens. This article identifies initiatives facilitating the civic participation of older people in policy-making in European Union member and associate states, drawing on a review of the literature, consultation of national policy experts, and exemplary case studies. Four main patterns of senior civic participation are identified: adopting consultative or co-decisional participatory approaches in policy design or policy implementation. The four are represented to varying degrees at different geographical levels (national, regional, local), with different actor configurations (appointed, elected/nominated, corporate representation), and with varying degree of institutionalization (temporary/permanent). Case studies illustrate approaches taken to enhance the quality and effectiveness of public services for senior citizens. Future research should strengthen this line of enquiry to cast further light on conditions facilitating the civic participation of senior citizens.

## 1. Introduction

Perceptions of political and economic elites appropriating democratically elected governments are leading to growing citizen mistrust toward democratic institutions and representatives [1,2]. Participation in elections has declined worldwide, and in large parts of Europe [3], local, regional, and national governments have been urged to find new means for halting and reversing political disenchantment [4]. The current “democratic deficit” suggests that political elections ought no longer to be considered the sole source of democratic legitimacy [5].

Almost 30 years after the European Union (EU) Maastricht agreement, which sought to reduce territorial inequalities through solidarity and strategic policymaking, challenges for more active citizenship remain at the center of the European political debate. In the last twenty years, the shift toward new forms of expressing public interest as a responsibility of government through the engagement of civil society has been a key topic for international and transnational agencies [6,7,8]. As Arnstein [9] put it, citizen participation should help progress toward real power of decision through the redistribution of information and resources, and the possibility effectively to influence policy-making.

Scholarly debate has explored the positives and negatives of constituting wider policy networks with social actors and stakeholders. The mainstreaming of citizen participation led by international and transnational agencies resulted in debates on how to strengthen legitimacy and trust in democratic institutions [6,8,10,11]. The literature associates citizen participation with civic values and democratic institutions, providing a greater sense of freedom and control, feelings of political efficacy, and sense of belonging to participants. In contrast, participation is often criticized for being too expensive and slow, and based on a false notion of democracy [12]. However, this argument overlooks that interest groups and their participation are vital contributors to political systems, even as we acknowledge that not everyone wants or has the resources to participate in civic debate [13].

### Civic Participation in the Literature

The prospect of a rapidly aging population in Europe has alerted the EU and member states to consider new forms of civic engagement [14,15,16,17]. In recent decades, older people have benefited from improved economic conditions, social protection, and health services in western democracies, which helped increase longevity and well-being. Yet ageism (e.g., multiple forms of stigmatization in the labor market [18]), marginalization (e.g., loneliness and social exclusion [19]) and poverty continue to adversely affect many senior citizens [20,21,22,23].

Whereas senior citizens show high levels of participation in political elections [21,24], a sense of powerlessness and disappointment with traditional politics may discourage them from engaging in forms of collective action due to, among others, ageist structures in society [25,26,27]. Against this backdrop, policy agendas on active aging have sought to improve public health, participation, and social security as drivers to enhance overall quality of life [28], supported by growing evidence that participation has a positive influence on health [29,30]. Strong emphasis has been placed on improving the well-being of senior citizens, participation in the labor market, promotion of cultural and social activities, as well as volunteering in a wide range of initiatives that generate (unpaid) social value [31,32,33].

The call for active aging in contemporary societies has had global reach [34,35], and a special case has been made for greater involvement of older people in political processes directly affecting them [23,24,28]. Participation can benefit the well-being of seniors [36,37], improving the quality, effectiveness, and efficiency of public measures, and boost innovative solutions [38,39], especially with regard to social care and pension provision [25]. In an attempt to collect and review international contributions on the topic, Raymond et al. [40,41] and Levasseur et al. [42] have produced inventories of the multiple meanings and uses of the concept of senior citizens’ participation. More recently, Pinto and Neri [43], and Serrat et al. [26] equally provided an overview of the multiple meanings and practices of civic participation. The authors agree that participation can be developed in both individual and collective settings, and that outputs generally determine better quality of life and well-being. In particular, Pinto and Neri [43] argue that participation is associated with lower risks of morbidities, disability, and cognitive decline. Serrat et al. [26] find that senior citizens’ participation is associated with better physical and mental health, higher cognitive function, increased physical activity, and decreased loneliness. Thornton [44] further highlights that participation in social and political life can increase self-confidence, instill a sense of control in senior people, and lead to improved access to public services, often by encouraging cultural changes in organisations involved in the participatory processes. Moreover, scholars argue that seniors hold the potential to impact democratic policy-making as they are assigned, and enabled with, new roles in social activities [42] and forms of socio-political involvement and activism [40,41].

Predictors of, and barriers to, senior citizens’ participation have been at the center of the scholarly debate as the ability to participate is often defined by time constraints, personal capacity, and skills that facilitate a greater commitment to public engagement [45,46]. Pinto and Neri [43] argue that culture, beliefs, habits, and the concrete opportunities to engage in the place of origin makes significant differences in senior citizens’ participation. Serrat et al. [26] further point out that along with personality variables and motivations, opportunities given by the social environment are determinant, as in the case of longer participation in programmes and organisations. Social knowledge and communicative competences are also good predictors of participation [47], while limited access to resources, such as time, money, language barriers, perceptions of powerlessness, lack of stamina and persistence, alongside barriers to institutional accessibility [40,41], adversely affect opportunities for participation in all policy fields. Further exacerbating conditions may be environmental factors, from adverse climatic conditions and pollution to inadequate street lighting or pedestrian infrastructures, which together limit the scope for safe participation in community activities [48]. Studies have also shown that participation in public activities can differ according to the health status of senior citizens. For instance, people with mild or severe health problems, or depressive symptoms are less likely to be involved in volunteering, while high income is associated with a higher propensity to volunteering in the case of mild health problems [33]. Principi et al. [33] also find that being widowed and suffering from health issues are positively associated with volunteering. In contrast, other scholars find that older people who are married often perform more social activities than those living alone [49]. Bukov [45] notes that participation is highly gendered—with men more likely to be engaged in political activities and clubs, and women in volunteering and caregiving—and biased toward higher socioeconomic status. Pinto and Neri [43] add that men tend to disengage from political and organisational activities, while women discontinue community activities and volunteering, especially due to widowhood, retirement, health problems, and functional decline. To avoid social exclusion and the civic discontent it may entail, scholars agree on the need to train public staff in participatory policy-making and improve communication with older populations [22,36,44,50,51].

In some cases, the actions of grassroots groups have contributed to a better understanding of the role that senior citizens can play in civic matters [39]. In tandem with informal initiatives, the growth of participatory initiatives with senior citizens at different levels of governance has also captured political authorities and corporate bodies at the international level [52,53,54,55,56]. The Vienna International Plan of Action on Aging [57], one of the most relevant plans in this field endorsed by the United Nations (UN), underpins the need for participation in community organisations and political processes. Likewise, the Principles for Older Persons issued by UN in 1991 argue the necessity to participate actively in the formulation and implementation of policies that directly affect seniors’ well-being [58] and share their knowledge and skills with younger generations. More recently, the Madrid International Plan of Action on Aging advanced the idea that measures should be taken to enable the full and equal participation of older persons in decision-making processes at all levels [35].

While political participation of older people may take place in various ways, notably through organisations of older people (e.g., civic society organisations), by electing political representatives (e.g., voting), as well as engaging directly in decision-making (e.g., consultative and co-decisional participatory initiatives) [59], research on the latter case has been limited. Political activity through organisations and electoral voting is highly practiced by older people and documented in research, but much less is known about opportunities and models for senior citizens’ civic participation in consultative and co-decisional initiatives [27,59]. This study seeks to narrow our knowledge gap.

Statutory bodies and elected representatives may show resistance to sharing or indeed relinquishing the power of decision-making [60]. Yet the call for action against poverty, economic decline, or rural remoteness [61] can persuade governments to seek support from organisations representing senior citizens’ interests in a range of policy domains. This presents these organisations with new opportunities for advocating for senior citizens’ needs, promoting measures and initiatives in support of active, participatory aging, as well as organizing participatory settings, such as forums, networks, user panels, day centers or care home user groups, or services planning groups [62,63]. According to the UN [64], organisations of older persons provide an important means of participation through advocacy, particularly for otherwise voiceless senior people, and of influence over decision-making processes at all levels of government. At the same time, scholars alert to the need for representativeness of seniors’ voice to avoid reinforcing social biases. Communicatively competent senior citizens, who are more frequently engaged in participatory initiatives, may not speak for or on behalf of other seniors, but rather represent personal or sectarian agendas [44,65]. Studying the relationship between senior citizen organisations and governmental agencies, Carter and Beresford [66] note that processes that are led by agencies and representative organisations may be more likely to gain official credibility and funding, although they may also be more likely accountable to, and constrained by, their own agendas. In contrast, initiatives that are led by individuals who do not identify themselves with any specific organisation can have more credibility, even if they often face greater obstacles in raising funding [21,46,67].

Carter and Beresford [66] further distinguish between consultative (or consumerist) approaches to senior citizens’ participation, which commonly aim to improve existing services as in the case of senior citizens’ councils and forums; and co-decisional (or democratic) approaches, which share power over decisions on issues of public interest. The latter range from individualized initiatives led by senior citizens (e.g., direct payment-based schemes), to social groups, networking, campaigning and direct action (e.g., civic forms, community councils, local area forums, committees and citizens’ panels) [63,67,68]. Overall, consultation is limited to the collection of citizen opinions about a specific issue and frequently results in low-profile deliberation, while co-decisional approaches set the conditions for a deliberative dialogue between governors and governed [44,66,69].

In both approaches, the influence of senior citizens depends on the stage of the policy cycle in which seniors are engaged [24,66,70,71]. Scholars tend to agree that participation should not be limited to isolated instances or individual stages of policy-making, but rather be embedded from the policy design to the end stage of implementation. According to Cooper et al. [72], citizen participation can be developed in either stage of the policy cycle, but that holds different potential for achieving public involvement. Whereas participation in the design of policies is expected to shape citizen opinions on policy topics and develop a common language for discussing problem definitions, policy implementation is likely to provide shared resources to co-produce the services to be delivered [73]. The distinction between the two stages builds on a longer debate among policy analysts on definitions of goal setting and their realization in action. Scholars have recently highlighted the path-dependent character of policymaking and the decisive impact of citizen participation in both stages, as policy design and implementation equally concern the generation of inputs through debate and activity [74].

The decision to either adopt a consultative or a co-decisional approach, then, should take into account policy stages as entry points to understanding participatory initiatives rather than as distinctly separated moments of policy-making. While participatory processes offer a unique cross-section of how different approaches can be adopted in different policy stages, the main body of literature points to additional analytical layers, such as: (i) the different and sometimes multiple scales of implementation [21,24]; (ii) the configuration of actors within the participatory setting, namely through senior citizens’ representatives appointed by statutory bodies, senior citizens’ organisations, and senior citizens and/or representatives directly elected by senior citizens themselves [47,65]; (iii) the degree of institutional embeddedness (i.e., permanent versus temporary involvement) of the participatory initiative in the governance system [66,75,76].

Acknowledging the need to systematise our knowledge of participatory initiatives engaging senior people in consultative and co-decisional policy-making (and, further, in public policy-making and delivery), this article describes and discusses examples of this kind of senior citizens’ *civic participation* in a subset of European Union member and associate states. This article is based on the 18-month European Commission-funded research project MOPACT. The whole project duration was 2013–2017 [77].

The following sections start with a description of the methodology adopted to identify emerging patterns of senior citizens’ participation in policy-making by layering gray and scientific literature with iterative collection of cases in Europe. Results are presented and discussed with the help of a sample of exemplary case studies selected to demonstrate and give empirical substance to the conceptual categories we identified from the review of literature [78]. The article ends with a summary of key findings and reflections on future research.

## 2. Materials and Methods

This study of senior citizens’ civic participation in Europe focuses on civic initiatives implemented in Europe between 2014 and 2015. Data was initially collected by way of a review of gray and scientific literature on the topic, using tools, such as Google Advanced, PubMed and OpenGrey, using combinations of keyword searches, including but not limited to “active aging”, “political participation”, “engagement”, “consultation”, “senior”. Participatory initiatives were further identified in reports on active ageing by international agencies (e.g., Age Platform, the European Union/Commission, the United Nations and the World Health Organisation). International networks on participatory policy-making, such as “Participedia”, “Open Democracy”, and the “International Observatory for Participatory Democracy”, were also included in the search.

Building on the classification by Carter and Beresford [66] of consultative or co-decisional approaches, the study draws insights from expert policy analysis [72,74] and distinguishes further between policy design and policy implementation stages (see Table 1). Policy design is here understood as the stage that defines how policy agendas are set and what policies should be documented as binding (e.g., laws, white papers, plans), and policy implementation as the stage that delivers public services.

Data collection was broad in scope, before case materials were assigned to the above matrix (Table 1). The retrieval of data guided the subsequent validation [79]. To validate the data, we consulted experts who had previously acted as National Coordinators of the 2012 European Year of Active Aging and Intergenerational Solidarity (EYAA), appointed by each of the 27 EU member states at the time and three associated countries (Norway, Iceland and Lichtenstein), and typically representing national umbrella organisations concerned with aging issues and supporting senior citizens. During the 2012 EYAA, the European Commission promoted active aging initiatives coordinated by National Coordinators in each member state. The Coordinators and the persons/organisations directly involved in identified initiatives were approached via email, and invited to verify initiatives and provide any additional information about practices in their respective countries. They were thus well positioned to be familiar with the types of initiatives our study sought to identify. These consultations helped to identify new, and to confirm the nature and ambitions of known, initiatives. Additionally, we approached researchers from across Europe to help us identify senior participation initiatives.

The results from the enquiries were collected and organized by the research team. Expert consultations confirmed and secured further details on 37 [77,80] out of 80 initiatives originally identified through literature and web searches. Forty-three initiatives were discarded as they had either been misidentified, or because no substantive information could be obtained from official sources (e.g., websites) or experts. A list of these 43 initiatives may be obtained from the authors upon request. In two instances, initiatives were merged into one as they originated from within the same legislative framework. This was the case for 11 Regional Councils for senior citizens in Spain, whose constitution had been mandated by the State Council of Senior Citizens, a public consultative body under the Spanish Ministry of Health set up in 1994 (see Appendix A. The second instance concerned two senior citizens’ representative regional bodies in Germany, which had been set up under a Federal Government Programme, ‘Active in Old Age’ (see Appendix A).

Initial analysis of the data suggested a diversity of initiatives that could be better captured by intersecting the two original coordinates based on the different participatory approaches and policy stages with three additional layers of analysis (Table 2).

First, we found that the political (and associated geographical) level at which participatory initiatives are implemented helped to understand their potential for influencing policy-making. Following Walker [24] and Goerres [21], initiatives in Europe can be classified according to the local (micro), regional (meso) or national (macro) levels. Macro- and meso-levels frequently have an instrumental role for improving service delivery at the local level, typically in the health and social care domains. Walker [24] further stresses that whereas the national level is more likely supported by “representative” bodies that provide guidelines for levering and driving local “person-centered” experiences, the meso-level increasingly shows the mushrooming of new action groups.

Second, we observed senior citizens’ representation in policy-making was either direct or mediated. In the latter case, seniors are represented by statutory bodies and/or organisations that are mandated to negotiate final decisions with public authorities [24,38,61]. Accordingly, we distinguished between configurations of actors in the participatory settings through the direct engagement of senior citizens (or their self-representation through nomination/election); the involvement of representatives typically appointed by governments; and the participation of senior citizens’ organisations. In the first case, senior citizens are given the right and opportunity to have a direct say, either in person or via a chosen spokesperson. In the second and third cases, senior participation is developed via proxies as representatives speak on behalf of senior citizens, be they politically appointed (e.g., in councils and forums) or seconded from the third sector [47,62,63,81].

Third, we explored the status of the participatory initiatives within the system of governance to capture information about their embeddedness within the policy agenda. Our key indicator is the policy initiative’s intended or expected duration, which is often reflected in legal frameworks [75,76]. Time-limited and single-purpose initiatives tend to be subject to electoral cycles and vulnerable to political whim [60,66,82], to which statutory initiatives are less likely to be subjected. We thus observe degrees of institutionalization, distinguishing between permanent initiatives relying upon legal frameworks and temporary initiatives with status- and time-limitations.

## 3. Results

The study allocated the 37 confirmed senior citizen participatory initiatives within the two coordinates described above: participatory approaches (consultation and co-decision) and policy stages (policy design and implementation). As illustrated in Table 3, the combination of these coordinates results in four types of participatory initiatives that were identified in the European member and associated states: 28 consultative initiatives in policy design; six consultative initiatives in policy implementation; two co-decisional initiatives in policy design; and one co-decisional initiative in policy implementation.

Initiatives adopting consultative approaches outnumbered co-decisional approaches, with only three co-decisional initiatives identified in this study. Efforts to provide senior citizens with opportunities to determine resource allocations appeared to be comparatively rare, albeit not without examples. Likewise, fewer participatory initiatives sought to provide senior citizens with opportunities to have a say in the implementation of policies and the delivery of public services [69], as most sought to facilitate involvement in policy design [75]. Additionally, as described above, we analyzed cases according to three further layers of analysis, which helped to understand scales of implementation or reach, configuration of actors in the participatory settings, and degrees of institutionalization (Table 4).

The majority of civic participation initiatives are implemented at a local (=17) or a national (=15) scale. They are largely single-site projects, but also include initiatives implemented in multiple locations under a common legislative framework. Participatory initiatives tend to involve senior citizens directly in policy-making, including 19 initiatives via delegated or elected representation of senior citizens in both service design and delivery. A further 11 practices seek to ensure representation of senior citizens by way of appointment of senior representatives to public policy boards; while 7 initiatives involve senior citizens indirectly through advocacy organisations. Most initiatives are permanent, anticipated to continue their work for the foreseeable future, often with funding formally secured—or confident of securing it—for some years ahead. Two of the three temporary initiatives (“Streets are ours also” in Lisbon, Portugal; and “Mobility and safe streets: older generations in movement” in Rome, Italy) share a narrow policy agenda with a focus on urban interventions in streets and public spaces. The third temporary initiative, involving Black and Minority Ethnic seniors in the United Kingdom, was set up to trial participation of socially and politically excluded minority populations in 2009, but did not progress to full implementation.

Table 5 lists the 37 initiatives according to the above coordinates. Web links to these initiatives, as retrieved at the time of the study, can be found in Appendix A.

### 3.1. Characterization of Emerging Patterns

As shown in Table 3 and Table 5, few initiatives adopted a co-decisional approach. Solely for the purpose of describing co-decisional initiatives, the three case study examples are merged into one combined grouping (or pattern). To illustrate the distinctive features to each of the (now three) principal patterns, we include three exemplary case studies for each. As Flyvbjerg [78] put it, case studies have capacity to highlight general and generic characteristics and help to set standards of understanding differences. Our data assembly and presentation are necessarily reflections of the theoretical positioning that took shape during our empirical work as we consulted multiple scientific and gray sources [79].

#### 3.1.1. First Pattern: Participatory Initiatives Adopting Consultative Approaches in Policy Design

Participatory initiatives in policy design are mainly situated in councils and forums that rely on consultative arrangements for gathering information and opinions with and from senior citizens. These statutory bodies may operate at multiple levels, often cross-cutting scales, and their aim is to facilitate dialogue between senior citizens’ representatives, elected and public officials, and other stakeholders. Government consultation on policies affecting senior citizens is, in several cases, mandatory and regulated by national legislation. In some instances, such arrangements involving councils and forums resulted from the actions of senior citizens’ organisations, persuading and encouraging public authorities to enshrine participation by regulatory means.

The three examples below are illustrations of consultative mechanisms of different origins and different statutory settings: the Senior Citizens’ Councils in Denmark, the Senior Citizens’ Council in Dortmund (Germany), and the Older People Councils in Ireland. As previously argued by Carter and Beresford [66], Thornton [44] and Walker [24], legal foundations and financing mechanisms simultaneously define the influence that these organisations have on political decision-makers. In all three instances, national guidance or, as in the Danish case, legislation and oversight, further seek to ensure that representations achieve appropriate gender and ethnic equality. In the Danish case, for instance, inclusion of women is encouraged via expense allowances supported by the municipality via an expense allowance that can reach €73 per month for older women, plus a budget of €6000 a year to use for the organisation of the councils’ activities. In the Irish case, Councils are committed to preventing socioeconomic bias in the representation of senior citizens’ needs and interests by including the most vulnerable groups [40,41,61]. Moreover, the direct election of senior citizens’ representatives in the Danish and the German cases, is understood locally to contribute to their status and their capacity to shape public policy.

The Senior Citizens’ Councils in Denmark (SCCs) is one example of local initiatives that resulted from national legislation, here issued in 1996, which mandated local municipalities across Denmark to set up and consult with their local SCCs, directly elected by senior citizens aged 60 or older. Members are prevented from assuming political functions other than advising on policy matters that most directly concern senior citizens (e.g., primary health care, social policies, local infrastructure). At the end of the 1990s, the SCCs gave impetus to the creation of the National Association of Senior Citizens’ Councils (Danske Ældreråd). The SCC comprise about 1000 members in total throughout Denmark, with each SCC composed on average of 10 members. Senior citizens can run as candidates to represent senior citizens’ interests and, to-date, the elections have returned about half of the existing SCC membership with the other half being newly elected.

The Senior Citizens’ Council in Dortmund (DSCC), in the Land of North Rhine Westphalia in Germany, is a local permanent initiative for senior citizens. In contrast to the above-mentioned Danish case, no national statutory law regulates senior citizens’ councils in Germany, as federal law delegates responsibility for social policy to the regions (‘Länder’). The DSCC came about as the result of grassroots pressures and was composed of representatives of the welfare organisations before the City Council recognized it as a municipal constituency in 1994. Like the SCC in Denmark, senior citizens’ representatives of the DSCC, 27 in total, are now also directly elected by city residents aged 60 and older. The representatives then participate in municipal committees responsible for social and health care, housing, culture, sports and leisure time, and public relation.

The Older People Councils in Ireland (OPCs) builds on Ireland’s National Positive Aging Strategy (2011–2016), a national framework for local initiatives with appointed representatives of senior citizens forming statutory bodies. Unlike SCC and DSCC, OPCs are neither mandatory nor regulated by law. Under the umbrella of the National Positive Aging Strategy, local and regional coalitions are encouraged to set up executive steering groups with representatives from city and/or county councils, meeting every 6–8 weeks. Their terms last 2 to 3 years during which they elect representatives onto the age friendly alliance. Each OPC holds an annual general assembly to report on progress and elect new steering groups if needed. At the time of this study, 18 OPCs had been established, including some going back to the late 1990s, prior to the declaration of the national strategy. Local authorities are tasked with ensuring that OPCs are representative of the diversity of the older population, especially of the most vulnerable.

#### 3.1.2. Second Pattern: Participatory Initiatives Adopting Consultative Approaches in Policy Implementation

The consultation of senior citizens for service delivery has typically evolved from governmental and legislative structures through the involvement of civil society organisations. Our study captured a current, but quite possibly ‘temporary state’ in the development of initiatives that operate similar to lobby groups. Organisations and alliances advocating for senior citizens are equally contributing to providing information and to enabling senior people to express service needs and preferences.

Three examples illustrate models of consultative approaches to policy implementation for and with senior citizens [66,70] by associative bodies [63,65]: the Partnership for Older People Programme in Dorset (England), the initiative called “A City for All Ages” in Edinburgh (Scotland), and the National Network Forum for Helping Older People in Slovakia.

Financial and human resources emerge as critical facilitators in all instances, and most notably in the Partnership for Older People Programme in England, which has access to grant-funded paid as well as volunteer personnel in order to support a program of activities enabling senior citizens to influence service delivery. The initiative in Edinburgh also benefits from direct funding, here provided by local government [60]. In contrast, the senior participation initiative in Slovakia has no resources to fund personnel on the ground and instead relies on negotiating and bargaining with public authorities for better services and facilities [51].

The Partnership for Older People Programme in Dorset, England, (POPP) is a local and permanent initiative that builds on the direct engagement of senior citizens. It focuses on participatory measures for senior populations in the provision of health and social care services. POPP staff argue that planning and management of the activities are fundamental to obtaining tangible outcomes and, thus, improve members’ satisfaction. Toward this aim, consultative approaches are adopted through the local Primary Care Trust (responsible for delivering health service and care in the area), and a set of other initiatives. The POPP Strategic Board is composed of four senior citizens sitting alongside three representatives from Dorset County Council and the National Health Services. It decides on funding proposals, while encouraging Council bodies to respond quickly to community needs. Evaluations suggest that the initiative has been cost-effective and has helped to improve service delivery by facilitating dialogue between senior citizens and professional service providers. Participation in the process of designing and implementing projects is via the Community Initiatives Commissioning Fund (open to not-for-profit local groups) and the Dementia Innovation Fund (open also to professionals). In 2015, the POPP budget had been set at £800,000 per annum.

In the Scottish city of Edinburgh, an initiative called “A City for All Ages” (ACFAA) addresses a wider set of policy areas than POPP’s health and social care focus does. Like the POPP, ACFAA is a local and permanent initiative that engages senior citizens directly. It holds a budget for staff and resources and operates within municipal institutions. The initiative seeks to reduce the risk of social isolation, associated depression, health risks (e.g., dementia) and poverty among senior citizens. ACFAA was created under the ten-year strategy launched by the Edinburgh City Council in 2000 (“Plan for Older People”), which established an advisory group, recruited from local forums, groups and voluntary organisations, to inform council policy through consultations, which have included sessions on housing affordability and accessibility, as well as sheltered housing.

The National Network Forum for Helping Older People in Slovakia (Forum), finally, is a national initiative established in the year 2000 through a network of around 350 civil organisations that aim to improve the delivery of services to senior people at multiple levels. Unlike the POPP, the Forum relies on its membership both for funding and shaping its agenda. Like the ACFAA, its goal is to influence government decisions by working with and advising local and central government, and the police. The Forum helped to set up the Slovak Parliament of Seniors, a group of representatives of organisations and clubs for retirees from across Slovakia, which meets at least once a year to explore opportunities for more active social and civic engagement of seniors.

#### 3.1.3. Third Pattern: Participatory Initiatives Adopting Co-Decisional Approaches in Policy-Making

While we found only a few cases of co-decisional approaches to senior citizens’ participation in Europe, they included examples of both policy design and policy implementation. Two of these initiatives-the Senior Citizens’ Participatory Budget (SCPB) in Alfândega da Fé (Portugal), and the combination of the civil panel and the participatory budget in Gdynia (Poland)—seek to engage seniors through direct voting and self-advocacy, whereas the Active Participation Centers in Andalusia (APC; Spain) engage senior citizens through member representation. These Centers provide resources for senior citizens to have a voice in the management and implementation of public measures, and representatives elected by service users are charged with the responsible and responsive management of health and social policies in the centers [25,39,83].

In contrast to consumeristic approaches to participation, the three case studies are examples of measures seeking to enable direct, pro-active participation of senior citizens [60,63,66,67]. Senior citizens-centered activities promoted through a civil panel are combined with participatory mechanisms in the wider community in Gdyina (Poland), while the SCPB in Alfândega da Fé (Portugal) is an example of senior citizens-centered allocation of public budget. In this small town of Portugal with some 5000 inhabitants, senior people have the opportunity to propose projects for funding, which are selected by a local council body of senior citizens charged with supporting and shaping the work of the city council. This initiative sets aside funds to support social facilities and programs specifically intended for the town’s older population, aware of the challenge of social exclusion [23,40,81].

The SCPB, a statutory permanent initiative, was originally proposed by the Alfândega da Fé City Council in 2014 with a view to encourage the direct participation of senior citizens through mechanisms allowing them to decide on a share of the municipal budget (€10,000). The SCPB adopted a representative rather than electoral model of engagement with senior citizens, with those aged 65 and older eligible to propose local initiatives. Given high levels of illiteracy among the local population, and the territorial dispersion and isolation in rural villages, the municipality decided to provide the Senior Citizens’ Council, which had been created in 2013, with power to select senior citizens’ proposals that should be funded through the SCPB.

As in the SCPB, the municipality of Gdynia (Poland) encouraged the direct participation of senior citizens in local affairs. Around 30% of Gdynia’s inhabitants are seniors and the municipality has been promoting activities to foster a stronger dialogue with this population. A civil panel of senior citizens was organized in 2013 and 2015 to collect opinions on social services and public spaces. This initiative served to promote knowledge and awareness that informed the implementation of the city’s participatory budget initiative. Unlike the SCPB, however, the Gdynia consultations were open to citizens of all ages, which was balanced by the civil panel giving preference to projects considered especially beneficial to seniors, such as public infrastructure and spaces improvements.

Whereas the SCPB and the civic panel in Gdynia seek to engage directly senior citizens in the formulation of public policies, the APCs in the Spanish autonomous region of Andalusia are an example of co-decisional approaches in service delivery that act through the mediated participation of older people through elected representatives. The region adopted a strategy of active aging and operates more than 3500 social centers that serve and support, among other social groups, senior citizens. In early 2015, APC serve about half a million members aged over 60. Besides providing dining services to its members, APCs organize social, cultural and leisure activities, offer training (e.g., in computing) and legal advice to senior citizens. APC members can take part in the General Assembly, which decides on these activities in consultation with the regional authority and local Senior Citizens’ Councils (which can also participate in the assembly, but without the right to vote).

## 4. Discussion

Involvement of senior citizens in policy matters affecting them directly builds on a policy discourse on active aging, which casts light on the need and the opportunities for increasing the quality of life of older people in a rapidly aging population [14,15,16,84]. This study surveyed the landscape of civic participation initiatives for senior across Europe. It found a diversity of initiatives promoting senior participation in policymaking, adopting consultative and co-decisional approaches [66] at different stages in the policy cycle [24,70]. These primary approaches intersected with different implementation scales, actor configurations, and degrees of institutionalization. Local initiatives benefiting from permanent legal frameworks were more prominent than other types of participation. A few of the initiatives identified in this study were purposely temporary in nature, or were led by appointed representatives or organisations without the direct involvement of senior citizens through some form of electoral representation. Three principal findings emerged from the study.

Firstly, senior participation in policy design, be it for the identification of problems or the setting of agenda and goals, more commonly relies on consultative approaches. Only a few initiatives had adopted co-decisional approaches, which limits our understanding on what can be considered the highest level of direct democratic involvement in the early policy stages, and whether there is political interest in breaking the ceiling of consultation. Non-binding consultation is often enshrined by law and/or framed within specific regulatory requirements or strategies, which may narrow discussion whenever norms create obstacles to more informal expressions of participation, but can make the initiatives more credible and durable [66]. In fact, we did not find evidence in Denmark or Germany that local governments were opposed to sharing decision-making processes with seniors [60,62,63]. Rather the intermediation of collective bodies was typically understood as a necessary mechanism for representing senior citizens’ interests, being aware of the risk of failing to represent seniors and reinforcing social and community barriers [44,65]. In some cases, as in Denmark, the direct election of members to statutory bodies proved an effective mechanism for engaging senior citizens through consultative approaches.

Second, local informants agreed on the positive contribution that senior citizen consultation in policy implementation and, especially, service delivery made to more efficient resource allocation. Cost-effectiveness is crucial when considering the different types of funding (e.g., state-funded, crowdfunded, etc.) of the initiatives, which influence the forms of accountability to members or electors. As Carter and Beresford [66] put it, credibility is associated with funding of representative organisations and increases the need for accountability. An organisation’s capacity to operate efficiently, especially if independently funded, enhances their capacity to advocate for members, as shown in Dorset. State-dependency for funding, at least in the perception of our case study informants, potentially risks undermining an organisation’s capacity to influence public policy agendas. Complex initiatives and networks, moreover, may require formal channels of collaboration with public authorities, which may limit their capacity to promote and scale up grassroots campaigns as emerged in Slovakia. That said, it is worth acknowledging that self-organized initiatives by individuals have been shown to face similar issues of credibility and fund raising [21,46,67].

Third, the participation of senior citizens through co-decisional approaches shows the importance of institutional arrangements adopted for civic participation to make individuals’ voices heard and influential. The cases presented suggest that city councils often privilege the direct engagement of individuals as a way to promote participation for and with people. Senior citizens can be encouraged to propose their ideas for public funding associated with other statutory bodies representing senior citizens’ interests, as in the cases of Alfândega da Fé (Bragança, Portugal) and Gdynia (Poland). This type of participation can build on specific needs of seniors’ engagement and aim to find solutions to disadvantaged living conditions, such as those linked to rural remoteness [61]. In a similar vein, the creation of initiatives that boost a critical mass of senior citizens to be channeled into participatory initiatives as the participatory budget, can equally proportionate sound results. Co-decisional approaches can be applied for the management of public structures and for the improvement of their services through the intermediation of citizens’ representatives, as shown by the APCs in Andalusia.

While our study reached across Europe and provides a cross-sectional overview of emerging patterns in the field of senior citizens’ civic participation, we acknowledge the limitations of our research. First and foremost, data collection was often impaired by patchy information, often due to the ‘low profile’ of participatory initiatives that are rarely promoted outside their specific environments. It is thus quite possible that our study has missed important examples of civic participation, including local or regional initiatives, which our searches had identified, but that neither our country nor our international experts could validate. Moreover, the study did not cover examples of failed, abandoned, or discontinued approaches to senior citizen participation. Finally, although country experts were asked to include evidence of relevant initiatives in their national or any other language, our searches were limited to reports and forms of publicity in languages accessible to the members of the research team (English, Italian, German, Spanish and Portuguese).

Notwithstanding its limitations, we hope our study will help to formulate policy directions and future research on senior citizen participation. Findings and lessons may be considered in light of the recent disruption of the Covid-19 pandemic that according to UNDESA [85] is likely to exacerbate negative stereotypes about senior people. The initiatives discussed in this paper illustrate efforts to overcome social exclusion and barriers to participation as often manifest, for instance, in the under-representation of women (SCC, Denmark) or lower socio-economic groups (OPCs, Ireland), the lack of voice given to marginalized populations, such as seniors in housing need (ACFAA, Edinburgh) and pitfalls of senior citizens’ engagement due to the territorial dispersion of often illiterate local populations in rural areas (SCPB, Alfândega da Fé). The initiatives presented here present potential models for addressing age discrimination and deep-seated inequalities through a stronger commitment to the participation in public policies that affect all people and seniors in particular.

A first and obvious lesson for policy is that consultative initiatives at the point of policy design can make a positive difference to meeting the needs of older people, especially if supported by legislation and/or (national) policy strategies. Being age-inclusive, initiatives can improve effectiveness when combining mechanisms of direct election within the statutory bodies or organisations representing seniors’ interests. Second, consultative initiatives in policy implementation can deliver tangible results. Offering age-adequate services, these initiatives may be provided with funding to increase their accountability to both seniors and the wider public. Third, co-decisional initiatives providing senior citizens with a say in decisions on service delivery also have the potential to positively affect inclusion of marginalized older people, facilitating social participation through better infrastructure and support systems.

## 5. Conclusions

This article describes the findings of an exploratory study on senior citizens’ participation in policy-making. We combined literature searches and case studies in Europe to provide a better understanding on different types of participatory initiatives, which we have described as four distinct patterns. At the outset, we defined two baseline coordinates that allowed us to collate evidence about different participatory approaches (consultative or co-decisional) and policy stages (policy design or implementation). Three additional analytic layers, namely the spatial level of implementation, the configuration of actors in the participatory settings, and the degree of institutionalization, helped to further differentiate between types of initiatives.

While being explorative, the present study suggests a prevalence of non-binding consultative approaches to senior citizens’ civic participation in Europe, with some initiatives gaining strength and influence through their incorporation in legal framework and secure, independent funding. The latter especially increased public accountability of the funded bodies. Although divergent social, economic, and political contexts define the European countries and their jurisdictions, the varying geographical levels at which the initiatives operated suggest scope for developing participatory models operable at different territorial levels, adapting to or indeed break the siloes imposed by statutory administrative and political boundaries. Moreover, we have described different modes of engagement with, as far as we can ascertain in the absence of robust comparative evaluation evidence, initiatives based on direct election by senior citizens arguably in the strongest position of influence. Last, initiatives are subject to change, some institutionalized and intended to last, others not. Their influence on matters of civic concern to seniors may well be determined by their legal and constitutional status, which shapes their visibility and legitimacy within existing governmental structures.

We believe that the four patterns found in this study can and ought to be more carefully assessed and refined to explore the potential transferability of the sample of existing initiatives, which could be broadened and updated, within and outside Europe, and subjected to critical performance reviews. Furthermore, future research should help to shed new light on the upcoming challenges of civic participation throughout and after the Covid-19 pandemic, in particular with respect to the effort to provide equal conditions for participation to all senior citizens.

## Figures and Tables

**Table 1:** Types of senior citizens’ participatory initiatives in policy-making according to the two coordinates on participatory approaches and policy stages.

	Policy Design	Policy Implementation
**Consultative approach**	Initiatives focused on policy design through consultative approaches	Initiatives focused on policy implementation through consultative approaches
**Co-decisional approach**	Initiatives focused on policy design through co-decisional approaches	Initiatives focused on policy implementation through co-decisional approaches

**Table 2:** Systematization of empirical knowledge according to two coordinates and three layers of analysis.

Coordinates	Layers of Analysis	Characterization
Participatory approaches	Scale of implementation or geographical reach	National
		Regional
		Local
	Configuration of actors in the participatory setting	Appointed representatives
		Senior citizens’ organisations (corporate/advocacy)
		Direct engagement (nominated or elected)
	Degree of institutionalization	Permanent
		Temporary

**Table 3:** Number of senior citizens’ participatory initiatives in policy-making, by type of initiative.

Type of initiative	Policy Design	Policy Implementation
Consultative Approach	28	6
Co-decisional Approach	2	1

**Table 4:** Number of senior citizens’ participatory initiatives in policy-making, by scale of implementation, configuration of actors, and degree of institutionalization.

Layers of Analysis	Characterization	Number of Initiatives
Scale of implementation	National	15
	Regional	5
	Local	17
Configuration of actors	Appointed representatives	11
	Senior citizens’ organisations (corporate/advocacy)	7
	Direct engagement (nominated or elected)	19
Degree of institutionalization	Permanent	34
	Temporary	3

**Table 5:** Senior citizens’ participatory initiatives in Europe.

			Policy Design Stage			Policy Implementation Stage		
			National Scale	Regional Scale	Local Scale	National Scale	Regional Scale	Local Scale
**Consultative approach**	**Permanent institutionalization**	**Appointed representative**	Government Council for Older Persons and Population Aging, CZ	Flemish Council of the Elderly, Flanders, BE	Senior Citizens’ Council, Lagos, PT			
			State Council for Senior Citizens, ES	The Scottish Older People’s Assembly SOPA, SCOT	Senior Citizens’ Council, Bratislava, SK			
			National Council for Senior Citizens, NO	Senior Citizens’ Council, Canton of Ticino, CH				
			Council on Seniors Affairs, LV	Forums on Aging, ENG				
			Federal Senior Citizens Advisory Council, AU					
			Federal Advisory Council for the Elderly, BE					
			Act for Elderly Care, FI					
			Regional Councils for Senior Citizens, ES (11 initiatives)					
			National Positive Aging Strategy, IE					
		**Organisation**	Pensioners Affairs Board, LT			National Forum for Helping Older People, SK		
			Senior Citizens’ representative regional bodies, DE (2 initiatives)					
		**Direct engagement**	The Pensioners Parliament, NI		Senior Citizens’ Council, Leipzig, DE			Shaping the future of old age agency, Arnsberg, DE
			Parliamentary Working Group for Older People, PL		Senior Citizens’ Council, Dortmund, DE			A City for All Ages, Edinburgh, SCOT
			Senior Citizens’ Councils, DK		Council of Senior Citizens, Oliveira de Azeméis, PT			Partnership for Older People Programme, Dorset, ENG
					Older People’s Council, Brighton and Hove, ENG			
					Senior Citizens’ Council, Chiari, IT			
					Forum of Senior Citizens, Santa Maria da Feira, PT			
					City Council budget consultation, Portsmouth, ENG			
	**Temporary institutionalization**	**Direct engagement**			BME Elders Engagement Project, East Midlands, ENG			“Streets are ours” campaign, Lisbon, PT
								Mobility and safe street: older generations in movement, Rome, IT
**Co-decisional approach**	**Permanent institutionalization**	**Direct engagement**			Senior Citizens’ Participatory Budget, Alfândega da Fé, PT		Active Participation Centers, Andalusia, ES	
					Senior Citizens’ Panel, Gdynia, PL			

AU = Austria, BE = Belgium, CH = Switzerland, CZ = Czech Republic, DE = Germany, DK = Denmark, ENG = England, LT = Lithuania, ES = Spain, FI = Finland, IE = Ireland, IT = Italy, LT = Lithuania, LV = Latvia, NI = Northern Ireland, NO = Norway, PL = Poland, PT = Portugal, SCOT = Scotland, SK = Slovakia.

## Appendix A

**Table A1:** Web links to the 37 selected participatory initiatives. For further information, see Falanga et al., 2015 [77].

Government Council for Older Persons and Population Ageing in Czech Republic [86]
State Council for Senior Citizens in Spain [87]
National Council for Senior Citizens in Norway [88]
Federal Senior Citizens Advisory Council in Austria [89]
Federal Advisory Council for the Elderly in Belgium [90]
Act for Elderly Care in Finland [91]
Regional Councils for Senior Citizens in Spain (11 initiatives) [92]
Age-Friendly Counties and Cities Programme in Ireland [93]
Pensioners Affairs Board in Lithuania [94]
Senior Citizens’ representative regional bodies in Germany (2 initiatives) [95]
The Pensioners Parliament in Northern Ireland (UK) [96]
Parliamentary Working Group for Older People in Poland [97]
Senior Citizens’ Councils in Denmark [98]
Flemish Council of the Elderly in Flanders (Belgium) [99]
The Scottish Older People’s Assembly SOPA (Scotland) [100]
Senior Citizens’ Council in Canton of Ticino (Switzerland) [101]
Forums on Ageing in England (UK) [102]
Senior Citizens’ Council in Lagos (Portugal) [103]
Senior Citizens’ Council in Bratislava (Slovakia) [104]
Senior Citizens’ Council in Leipzig (Germany) [105]
Senior Citizens’ Council in Dortmund (Germany) [106]
Council of Senior Citizens in Oliveira de Azeméis (Portugal) [107]
Older People’s Council in Brighton and Hove (England) [108]
Forum of Senior Citizens in Santa Maria da Feira (Portugal) [109]
City Council budget consultation in Portsmouth (UK) [110]
BME Elders Engagement Project in East Midlands (UK) [111]
National Forum for Helping Older People in Slovakia [112]
Shaping the future of old age agency in Arnsberg (Germany) [113]
A City for All Ages in Edinburgh (Scotland) [114]
Partnership for Older People Programme in Dorset (England) [115]
Session “Streets are ours also” in Lisbon (Portugal) [116]
Senior Citizens’ Participatory Budget in Alfândega da Fé (Portugal) [117]
Senior Citizens’ Panel in Gdynia (Poland) [118]
Active Participation Centres in Andalusia (Spain) [119]
No web address identified:
Council on Seniors Affairs in Latvia
Senior Citizens’ Council in Chiari (Italy)
Mobility and safe streets: older generations in movement in Rome (Italy)
